# Production system establishment and in vitro study of CD19/CD3ε bite antibody secreted from *Pichia pastoris*

**DOI:** 10.1038/s41598-025-21889-4

**Published:** 2025-10-30

**Authors:** Boram Kim, Kyu Tae Byun, Seung Hyeon Lee, So Yeong Cheon, Chan Gil Kim

**Affiliations:** Department of Biotechnology and Research Institute for Biomedical and Health Science (RIBHS), College of Biomedical and Health Science, Konkuk University, Chungju, 27478 Republic of Korea

**Keywords:** Bi-specific t cell engager (BiTE), Pichia pastoris, Acute lymphoblastic leukemia (ALL), chinese hamster ovary (CHO), Biological techniques, Biotechnology, Microbiology

## Abstract

Blinatumomab is an anti-cancer Bispecific T-cell Engager (BiTE) antibody drug approved by the Food and Drug Administration (FDA) for treating acute lymphoblastic leukemia (ALL). It targets CD3ε, which is expressed on T cells and concomitantly targets CD19, an antigen specifically expressed in B-cell leukemia. Blinatumomab-bound T cells are activated to secrete perforin and granzyme, which induce cell death in B-cell leukemia. Blinatumomab is produced in the Chinese hamster ovary (CHO) cell expression system in consideration of post-translational modification (PTM) and immunogenicity. Although the characteristics of CHO cells are similar to those of other mammalian cells, it has low yield and high production cost. In this study, we developed the yeast species *Pichia pastoris*-produced BiTE antibody targeting CD19 and CD3ε, herein called *P. pastoris* (p)-blinatumomab, which is in-house produced blinatumomab. Approximately 1.2 mg/L CD19/CD3ε-targeting BiTE antibody (p-blinatumomab) was produced from the *P. pastoris* by transformation with the pHIL-S1 plasmid vector encoding the sequence of blinatumomab, which was 2.4 times higher than that produced in the CHO cell expression system based on the previous study. Enzyme-linked immunosorbent assay verified that the antigen binding affinity of the purified p-blinatumomab protein for CD19 and CD3ε antigens was 1.86 and 1.43 times higher than that of the commercial blinatumomab, BLINCYTO, respectively. In addition, flow cytometry confirmed that p-blinatumomab had a similar antigen binding affinity to CD19- and CD3ε-overexpressing cells compared to the BLINCYTO. In conclusion, we suggest that *P. pastoris* is a useful system for expressing and producing BiTE antibody platforms for therapeutic drugs.

**Supplementary Information:**

The online version contains supplementary material available at 10.1038/s41598-025-21889-4.

## Introduction

Acute lymphoblastic leukemia (ALL) is a type of blood cancer that develops from the abnormal malignant proliferation and transformation of immature lymphoid cells, called lymphoblasts, in the bone marrow^1^. Instead of healthy lymphocytes, abundant lymphoblasts, including B- or T-lymphoid progenitors, located in the bone marrow, blood, and extramedullary organs, including the spleen and lymph nodes, can interfere with the proper immune response, resulting in bleeding, fatigue, and infection^1–3^. Although ALL commonly occurs in children aged 1–4 years and the incidence rate declines with advancing age, it tends to occur again in individuals above 55 years of age and arises mostly from the B-cell lineage^1,4^. The 5-year survival rate for patients with ALL aged over 50 years and children diagnosed with ALL was 25% and 90%, respectively^1^. However, the survival rate for relapsed/refractory B-cell ALL droped by 15–50% and 10% in children and adults, respectively^5^. Moreover, regions with a low sociodemographic index (SDI) have a high mortality rate for ALL^6^.

Among the current strategies for ALL treatment, bispecific T-cell engagers (BiTEs) show promise for cancer immunotherapy^7^. BiTEs, bispecific antibodies (BsAbs), are target molecules that connect T cells and tumor-specific antigens to eradicate cancer^8^. Blinatumomab is the first BiTE anti-cancer antibody drug approved by the Food and Drug Administration (FDA) in 2014 for treating ALL^7,8^. It eliminates cancer by targeting cluster of differentiation (CD)3ε, a surface antigen on normal cytotoxic T cells, and CD19, which is expressed on the surface of B-cell lymphoma, allowing patients’ own T cells to attack malignant B cells^5^. Structurally, blinatumomab is an engineered fusion protein, composed of two single chain variable fragments (scFvs) that specifically bind to each antigen, CD19 and CD3ε, respectively, and are recombinantly connected by flexible glycine–serine (G4S) linker^7,9^. A flexible linker and specificity of antigen-binding domain are also responsible for its anti-cancer efficacy^9,10^.

The Chinese hamster ovary (CHO) cell line, a mammalian cell line commonly used for human therapeutic protein production, is used for producing blinatumomab^10,11^. CHO cells provide recombinant proteins with non-immunogenicity, high quality, and compatibility^12^. CHO cells typically undergo human-like post-translational modifications (PTMs), such as glycosylation; however, blinatumomab was designed as a non-glycosylated protein and thus has a reduced molecular weight (54 kDa)^11–13^. Meanwhile, recombinant protein production from CHO cells is extremely expensive cost owing to poor cell growth and low productivity^14^. Moreover, the culture medium for maintaining CHO cells contributes to high manufacturing expenses, and a prolonged culture period leads to poor yields and low quality of the recombinant proteins^15^. To address these limitations, alternative antibody production systems using plants, bacteria, insects, and yeast have been investigated^16^. Among them, yeast typically produces proteins up to 2–5 g/L after 5 days of fermentation in the absence of endotoxins^17^. Particularly, the yeast *Pichia pastoris* (*P. pastoris*) possesses the ability to release recombinant proteins into the culture medium, which results in higher spatiotemporal yields for proteins with lower structural complexity than that of CHO cells^18,19^. The yeast *P. pastoris* is utilized under relatively simple growth media and conditions^20^. Moreover, the *P. pastoris* expression system provides therapeutic proteins at a comparatively low price and with time efficiency^21^.

Therefore, in this study, we established a *P. pastoris* platform for producing BiTE antibodies, such as blinatumomab. Similar to the CHO cell expression system, the methylotrophic yeast *P. pastoris* expression system released recombinant proteins outside the cells, the cost of the medium was low, and the protein expression was easily induced by methanol (MeOH). In addition, the *in vitro* antigen-binding ability of our in-house produced anti-CD19/CD3ε BiTE antibody, herein referred to as p-blinatumomab, was similar to that of commercial blinatumomab BLINCYTO. In addition, p-blinatumomab specifically targets CD9- and CD3ε-expressing cancer cells, similar to BLINCYTO. The therapeutic protein production platform that we established could be helpful to manufacture various antibody pharmaceuticals at a low cost and with high productivity.

## Materials and methods

### Strains and plasmids

*Escherichia coli* DH5α was used as a host strain for plasmid vector propagation (Thermo Fisher Scientific, Waltham, MA, USA). *P. pastoris* GS115 (*his4*) and KM71 (*arg4*, *his4*, *aox1*::*AGR4*) (Invitrogen, Waltham, MA, USA) were used as hosts for expression and purification of p-blinatumomab (Anti-CD19/CD3ε BiTE antibody). The pHIL-S1 plasmid (Invitrogen) was used as the backbone vector to construct and clone the plasmid vector.

### P-blinatumomab expression plasmid vector construction and cloning

The blinatumomab BiTE antibody drug sequence (drug bank ID: DB09052; https://go.drugbank.com/drugs/DB09052) with arginine (R) amino acid added to the N-terminus, connecting the anti-CD19 and anti-CD3ε scFv sequences with a (GGGGS) linker, was codon-optimized for expression in *P. pastoris* strain. The target sequence was cloned between the XhoI and BamHI restriction sites in the pHIL-S1 plasmid. The insert sequence (Supplementary Information 1) was cloned between XhoI and BgIII (Isoschizomer) restriction sites. The primer sequences were 5’-GCA ATC TGT CTT CGC TCG AGA CAT ACA GTT GAC TC-3’ (Forward primer) and 5’-GAA CAG TCA TGT CTA AGG ATC TGA ATT CCT AAT GAT GAT GGT GG-3’ (Reverse primer) and each restriction enzyme sequence was written. The p-blinatumomab plasmid vector was transformed to *E. coli* DH5α competent cells and confirmed by DNA sequencing.

### P-blinatumomab-producing *P. pastoris* expression screening

For p-blinatumomab protein expression, yeast inoculum was placed in 5 mL buffered glycerol complex medium (BMGY) medium in a 50-mL baffled flask. and cultured overnight at 30 ℃ in a shaking incubator (250 rpm in a 50 mm orbital diameter)^22^, until the culture reaches an optical density (OD) 600 = 2–6 (log-phase growth, approximately 16–18 h). The OD 600 nm of all clones for cell density (~ 5 × 10^7^ cells/ mL) was checked. Harvest cells were centrifuged at 1,500–3,000×g for 5 min at 24 °C. The cell pellets were resuspend to an OD 600 of 1.0 in 15 mL buffered MeOH-complex medium (BMMY) medium for induction with MeOH (Final Conc. Of 0.5% MeOH, added every 24 h). After 96 h of MeOH induction, the culture was harvested by centrifuging at 3,500×g and 10 min and filtered through 0.22-µm filters. The culture supernatant was purified using Ni-NTA affinity chromatography resin (Cat #88222; Thermo Fisher Scientific). The Ni-NTA resin was pre-equilibrated with washing buffer (10 mM phosphate-buffered saline (PBS), pH 7.4, 0.05% Triton X-100). The culture supernatant was reacted with pre-equilibrated Ni-NTA resin for 3 h and washed three times with washing buffer; each p-blinatumomab proteins from different clones were eluted with 10 mM PBS (pH 7.4), 0.05% Triton X-100, and 200 mM imidazole. The eluted fractions were collected and analyzed using a 12% SDS–PAGE gel. The p-blinatumomab expression was visualized using Coomassie Blue R250. The concentration of the purified p-blinatumomab was determined by measuring the absorbance at 280 nm using a UV-Vis spectrophotometer. The final concentration was calculated using the molar extinction coefficient (ε280 = 121,170 M⁻¹cm⁻¹) and the molecular weight (54,250 Da) of the protein. The purification buffer was used as a blank. The final expression level was calculated as the total mass (µg) of purified protein recovered per liter (L) of culture medium and was expressed in units of µg/L.

### Production and purification of p-blinatumomab at the 1 L scale with GS115/p-blinatumomab #105 clone

The GS115/p-blinatumomab #105 clone was subjected to the aforementioned procedure for p-blinatumomab production. The GS115/p-blinatumomab #105 production clone was expanded in 100 mL of BMGY medium (30 °C, 250 rpm, diameter of 50 mm) for 24 h and shifted to 1 L of BMMY medium for induction. MeOH was added every 24 h to a final concentration of 0.5%, and cultivation continued for 96 h. The culture was clarified by centrifugation (3,000×g, 20 min, 4 °C) and filtration through a 0.22 μm membrane. The clarified supernatant was loaded onto a pre-packed Ni-NTA affinity column (Cytiva, Cat#17524701) previously equilibrated with 10 mM PBS (pH 7.4) using ÄKTA FPLC system. After sample loading, the column was washed with five column volumes of the same buffer, followed by an additional wash containing 25 mM imidazole to remove loosely bound contaminants. P-blinatumomab protein was eluted with 10 mM PBS (pH 7.4) and 200 mM imidazole. Pooled elution fractions were measured for absorbance at 280 nm, and analyzed on 12% SDS-PAGE gels stained with Coomassie Brilliant Blue R-250 to confirm the purity and integrity of the recombinant p-blinatumomab protein.

### LC-MS/MS analysis

To compare the sequence between p-blinatumomab and BLINCYTO (Amgen, Thousand oaks, CA, USA), p-blinatumomab was subjected to LC-ms/ms analysis and sequence matching using PEAKS Software (Bioinformatics Solutions Inc., Waterloo, ON, Canada). The expressed proteins were extracted from a 15% SDS–PAGE gel, cleaved using trypsin, and injected into the LC-ms/ms (Korea Basic Science Institute, Seoul, Korea). Detected peaks were matched to the BLINCYTO.

### MALDI-TOF MS

For comparison of the total molecular weight, p-blinatumomab and BLINCYTO molecular weights were measured using MALDI-TOF-MS analysis (MALDI-TOF/TOF 5800 system, Korea Basic Science Institute).

### Enzyme-linked immunosorbent assay (ELISA)

P-blinatumomab and BLINCYTO were analyzed for antigen-binding affinity by ELISA. They were subjected to Ni-NTA purification and serially diluted 350 nM final concentration to measure the *K*_d_. Human CD19 and CD3ε antigens were coated using by coating buffer (carbonate/bicarbonate buffer) at 4 ℃ overnight and then blocked with blocking buffer (1% BSA, PBS pH 7.4) for 1 h. BLINCYTO and p-blinatumomab were used as primary antibodies and incubated with human CD19 and CD3ε antigens at room temperature (15–25 °C) for 7 h. Anti-His tag HRP-conjugated mouse IgG antibody was used as a secondary antibody. After second antibody binding step, 100 µL 3,3′,5,5′-tetramethylbenzidine solution (Ultra TMB, Pierce, Cat# 34028, USA) was added and the reaction was stopped by adding 1 M HCl. The absorbance at 450 nm was measured using a spectrophotometer (Multiskan GO, Thermo Scientific, Waltham, MA, USA).

### Flow cytometry (FCM) analysis

The antigen-binding affinities of BLINCYTO and p-blinatumomab were analyzed using Raji (CD19-overexpressing cell line) and Jurkat (CD3ε-overexpressing cell line) cells. Both BLINCYTO and p-blinatumomab were purified using Ni-NTA resin, and their final concentrations were adjusted to 250 nM for CD19 antigen-binding affinity analysis and 1 µM for CD3ε antigen-binding affinity analysis. BLINCYTO and p-blinatumomab were serially diluted and used as primary antibodies to treat Raji and Jurkat cells. An anti-His tag FITC-conjugated monoclonal mouse antibody (Invitrogen, MA1-81891) was used as the secondary antibody.

### Statistical analysis

All statistical analyses were conducted using GraphPad Prism software (Version 8.0 or later, GraphPad Software, La Jolla, CA, USA). The dissociation constants (*K*d) were calculated using non-linear regression analysis. Quantitative data from *in vitro* experiments are expressed as the mean ± standard deviation (SD) based on three independent experiments (*n* = 3).

## Results

### P-blinatumomab design and construction from *P. pastoris* expression system

**Fig. 1:**
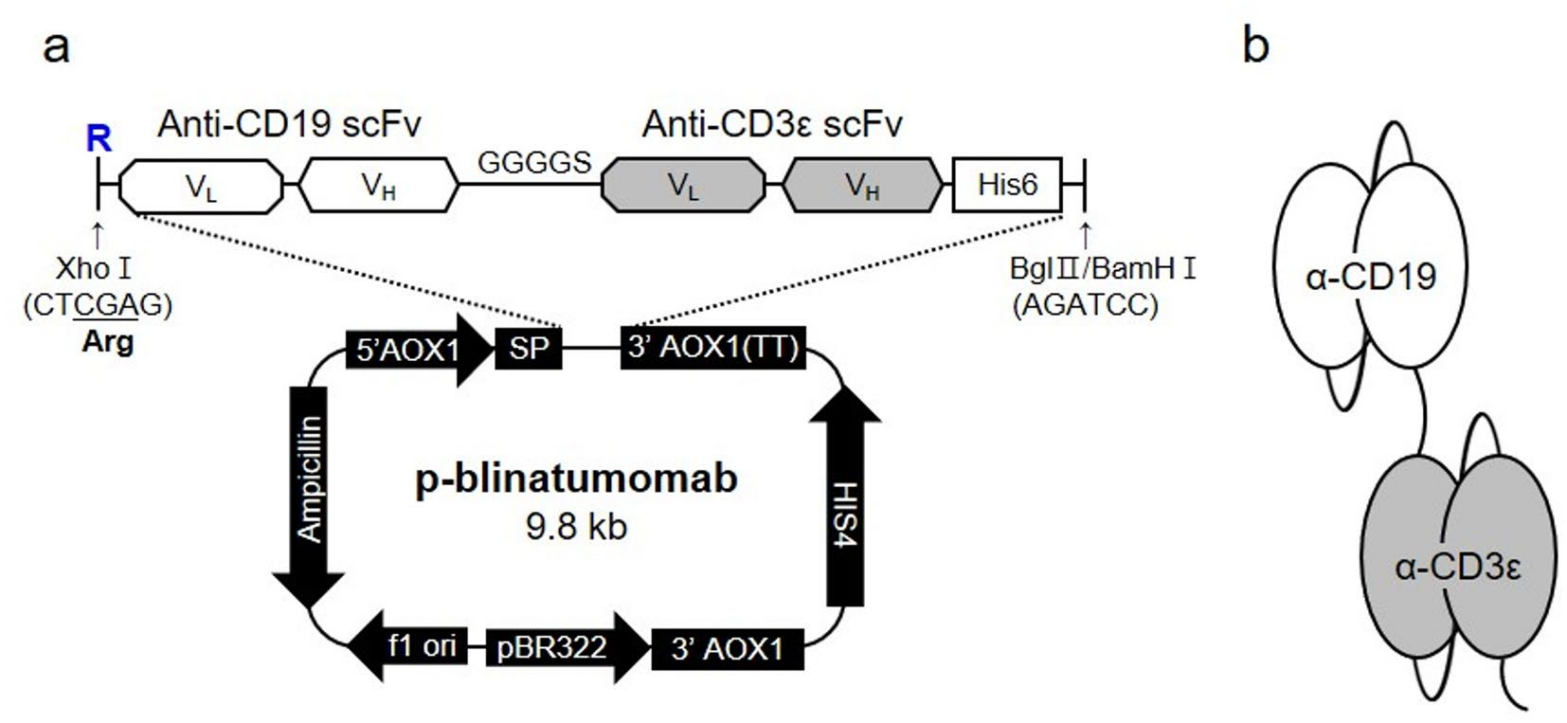
An in-house p-blinatumomab construction from *Pichia pastoris* expression system to produce blinatumomab. (**a**) The blinatumomab sequence was inserted in XhoI and BamHI enzyme cleavage sites of pHIL-S1 plasmid vector. p-blinatumomab BiTE antibody sequence transcript was added under the control of the *AOX1* promotor with hexa-histidine (6×His) tag. Arginine was added at the front due to the restriction enzyme site. (**b**) Schematic representation of p-blinatumomab domain structure.

The plasmid vector pHIL-S1 encoding p-blinatumomab was produced by transformation into *E. coli* strain DH5α. *P. pastoris* (yeast) strains GS115 and KM71, the most popular strains used as an important expression system particularly in industry and medicine fields^19^, were used for p-blinatumomab protein production (anti-CD19/CD3ε BiTE antibody) (Fig. 1). The p-blinatumomab sequence was constructed by linking the anti-CD19 scFv sequence with an arginine residue (R) added to the N-terminus and the anti-CD3ε scFv sequence with a GGGGS linker (all sequences in supplementary information). The anti-CD19/CD3ε scFv sequence was placed between the XhoI and BglII (BamHI Isochizomer) enzyme cleavage sites of the pHIL-S1 plasmid vector, thereby generating a p-blinatumomab sequence transcript tagged with hexa-histidine (6×His) under the control of the alcohol oxidase 1 gene (*AOX1*) promoter (Fig. 1a). Figure 1b shows the p-blinatumomab domains targeting CD19 and CD3ε antigen produced by *P. pastoris*.

### P-blinatumomab protein selection by small-scale screening

**Fig. 2:**
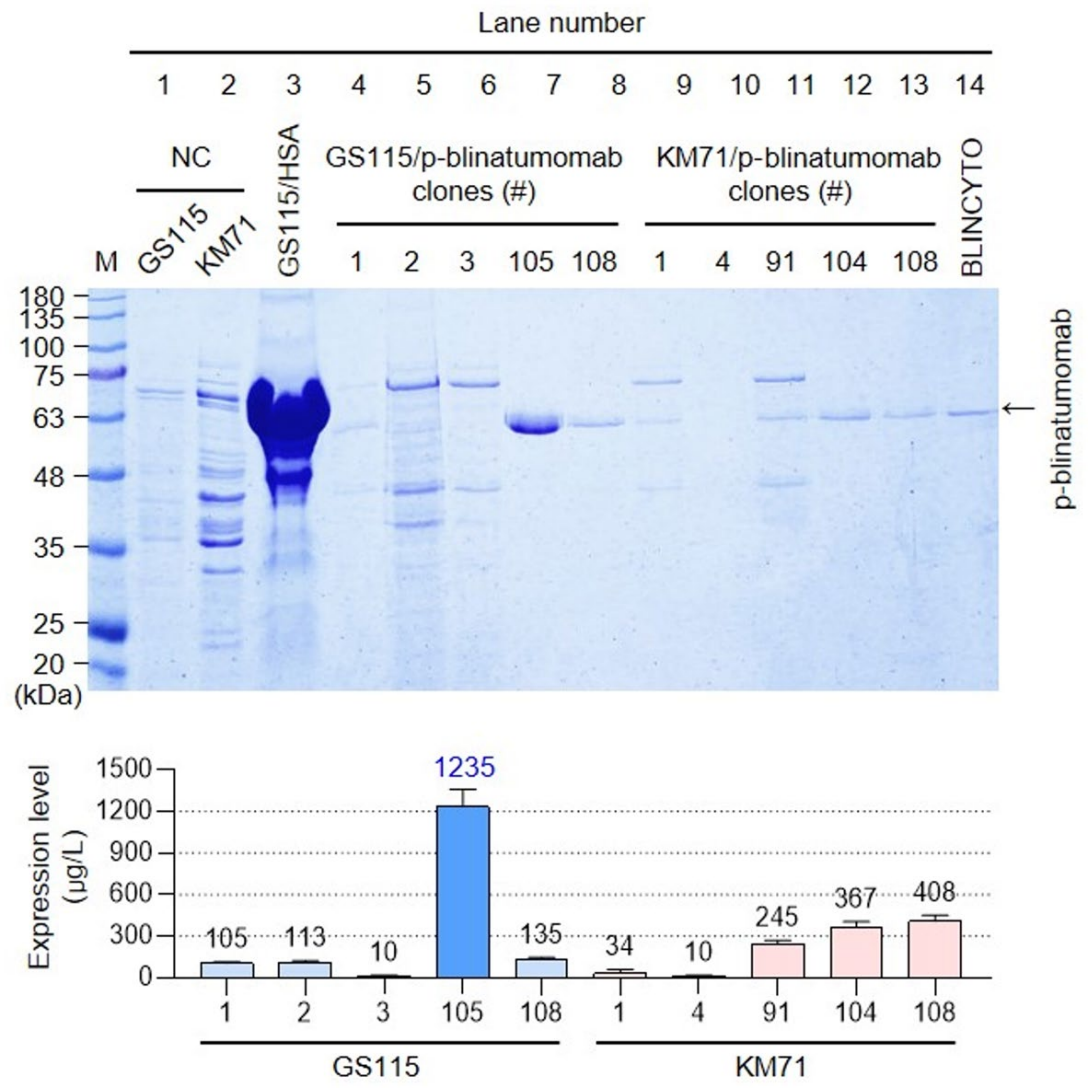
Small-scale screening of p-blinatumomab-expressing *Pichia pastoris* clone. (**a**) *P. pastoris* GS115 and KM71 clones transformed with the p-blinatumomab recombinant linearized plasmid DNA vector were cultured in BMMY medium with MeOH for 96 h (final MeOH concentration: 0.5%, 24-h intervals), and equal amounts (10 µL) of the extracted eluted fractions were analyzed on a 12% SDS-PAGE gel. Lane M, pre-stained protein marker (Kanpro Research. KP-PR001); Lanes 1 and 2, not transformed GS115 and KM71 (negative controls); Lane 3, GS115-HSA secreting human serum albumin (HSA) to BMMY medium (positive control); Lanes 4–13, 5 clones of GS115/p-blinatumomab (Clone number;1, 2, 3, 105, 108 and 5 clones of KM71/p-blinatumomab (Clones 1, 4, 91, 104, 108) purified by Ni-NTA resin affinity chromatography and analyzed on a 12% SDS–PAGE gel. The GS115/p-blinatumomab #105 clone producing the highest amount of p-blinatumomab was selected for further studies. Lane 14, 100 ng of BLINCYTO. HSA protein molecular weight is 63 kDa. (**b**) The expression rate of p-blinatumomab, quantified by UV-Vis spectrophotometry at 280 nm was determined as the total recovered purified protein (μg) per liter of culture medium (μg/L). All values are expressed as the mean ± standard deviation (SD) based on three independent experiments (*n* = 3).

After using *P. pastoris* GS115 and KM71 expression systems, we performed small-scale screening to assess recombinant p-blinatumomab protein expression (Fig. 2a). The SDS–PAGE gel image shows that the transformed GS115 and KM71 cells were used as negative controls (lanes 1 and 2, respectively). GS115-secreted human serum albumin (HSA) from the BMMY media was used as a positive control (approximately 63 kDa; lane 3). Proteins from five GS115/p-blinatumomab and KM71/p-blinatumomab clones are shown in lanes 4–8 and 9–13 respectively. In lane 14, 100 ng BLINCYTO was loaded to assess its molecular weight (between 48 and 63 kDa). In this screening, the protein in lane 7 was strongly expressed as a band for GS115/p-blinatumomab #105. The production yield graph shows that GS115/p-blinatumomab #105 displayed the highest production yield at 1,235 µ/L at 280 nm using a UV-Vis spectrophotometer (Fig. 2b). Therefore, we selected clone GS115/p-blinatumomab #105 for further study. The selection was based on identifying the clone that exhibited the highest expression level of an intact, non-degraded protein band at the expected molecular weight (54 kDa).

### GS115/p-blinatumomab #105-produced p-blinatumomab optimization and purification

**Fig. 3:**
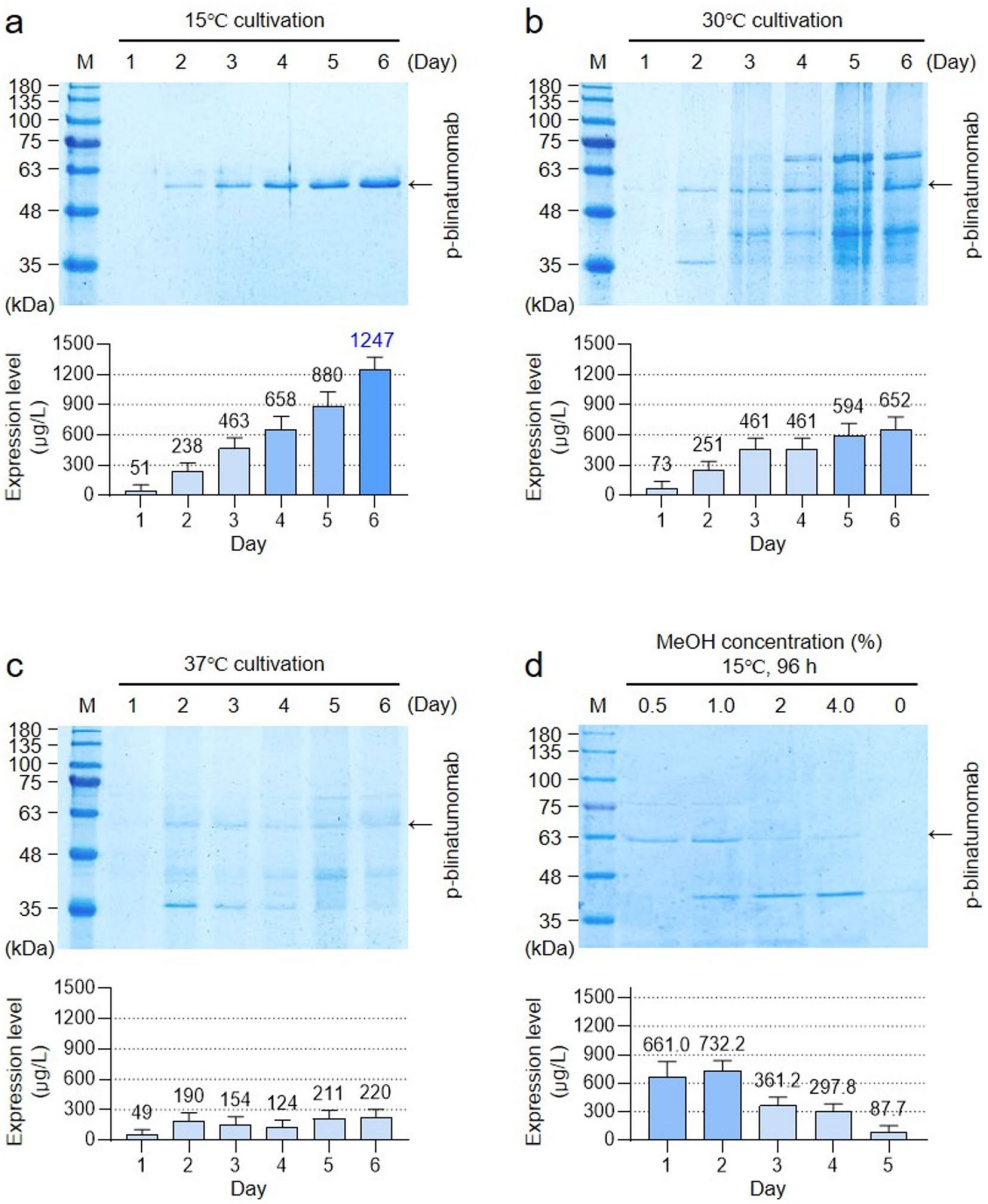
Optimization of GS115/p-blinatumomab #105-produced p-blinatumomab expression. (**a**–**c**) Cultivation at 15, 30, and 37 ℃ for 6 days (144 h). P-blinatumomab was monitored by 12% SDS–PAGE gel analysis. The bar graph numerically represents the expression level for each day using a UV-Vis spectrophotometer. (**d**) The p-blinatumomab concentration after growth at 15 ℃ for 96 h in the presence of different MeOH concentrations (0, 0.5, 1.0, 2.0 and 4.0%). P-blinatumomab was monitored by 12% SDS–PAGE gel analysis. The bar graph numerically represents the daily expression level of p-blinatumomab, quantified by UV-Vis spectrophotometry at 280 nm. All values are expressed as the mean ± standard deviation (SD) based on three independent experiments (*n* = 3).

**Fig. 4:**
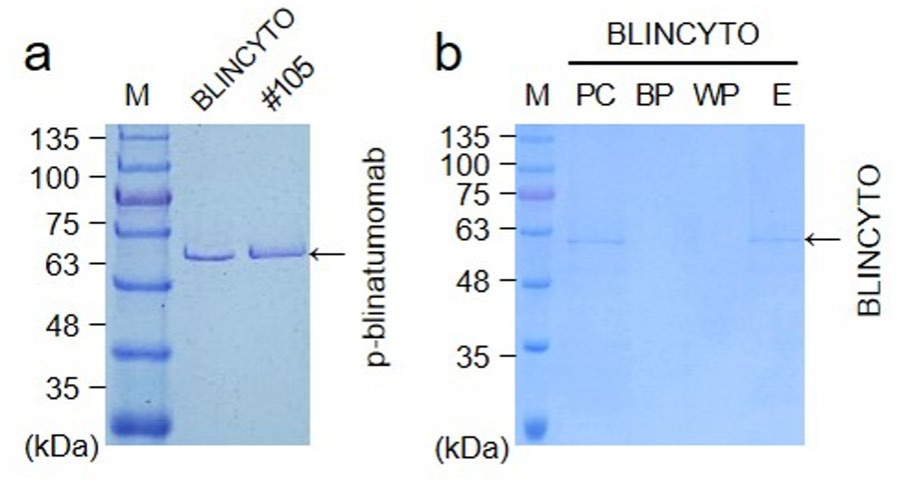
P-blinatumomab expression and purification from *Pichia pastoris*. (**a**) SDS–PAGE analysis of the p-blinatumomab expression and its subsequent purification via Ni-NTA resin affinity chromatography. The protein was separated on 12% SDS–PAGE gel and stained by Coomassie brilliant blue R250. Lane M, pre-stained protein marker (Kanpro Research. KP-PR001); Lane #105, p-blinatumomab induced by 0.5% MeOH for 96 h and purified by Ni-NTA affinity chromatography. (**b**) SDS–PAGE analysis of the BLINCYTO. Lane M, pre-stained protein marker (Kanpro Research. KP-PR001); Lane PC, 100 ng BLINCYTO; Lane BP, fraction of Ni-NTA affinity chromatography resin binding pass; Lane WP, fraction of Ni-NTA affinity chromatography resin washing pass; Lane E, fraction of Ni-NTA affinity chromatography elution.

Based on expression level (protein yield) and protein integrity and quality, we selected p-blinatumomab protein from *P. pastoris* GS115/p-blinatumomab #105 clone. To determine the optimal conditions for p-blinatumomab expression, we assessed p-blinatumomab expression under culture conditions at various temperatures and time points, and protein expression level was measured at 280 nm using a UV-Vis spectrophotometer (Fig. 3). At 15 ℃, the expression of p-blinatumomab was examined by SDS–PAGE for 6 days (Fig. 3a). The bands between 48 and 63 kDa corresponding to p-blinatumomab gradually increased in intensity over time. Likewise, at 30 ℃, p-blinatumomab levels steadily rise during 6 days (Fig. 3b). Although p-blinatumomab showed relative low expression at 37 ℃, it was maintained throughout the 6-day period (Fig. 3c). Figure 3d shows SDS-PAGE analysis of p-blinatumomab expressed at 15 °C in the presence of various MeOH concentrations (0, 0.5, 1.0, 2.0, and 4.0%). These data indicate that p-blinatumomab expression was maximized at 15 ℃ for 6 days, indicating that this condition is optimal. Through SDS-PAGE, unexpected bands were detected on the SDS-PAGE gels (Fig. 3b and c). Among them, immunoreactive bands were observed at approximately 75 kDa and 45 kDa, which are assumed to be alcohol oxidase (75 kDa) and mitochondrial alcohol dehydrogenase isozyme III (mADH), respectively^23,24^. Both alcohol oxidase and mADH are frequently detected during protein production and purification steps using *P. pastoris*^23,24^. Purified p-blinatumomab was obtained by Ni-NTA resin affinity chromatography, and its expression was confirmed by SDS–PAGE (Fig. 4a). The designated recombinant p-blinatumomab protein had an expected molecular weight (approximately 54 kDa). In addition, BLINCYTO was used as a positive control in SDS–PAGE (approximately 54 kDa) (Fig. 4b).

### Sequence concordance comparison between p-blinatumomab and BLINCYTO

**Fig. 5:**
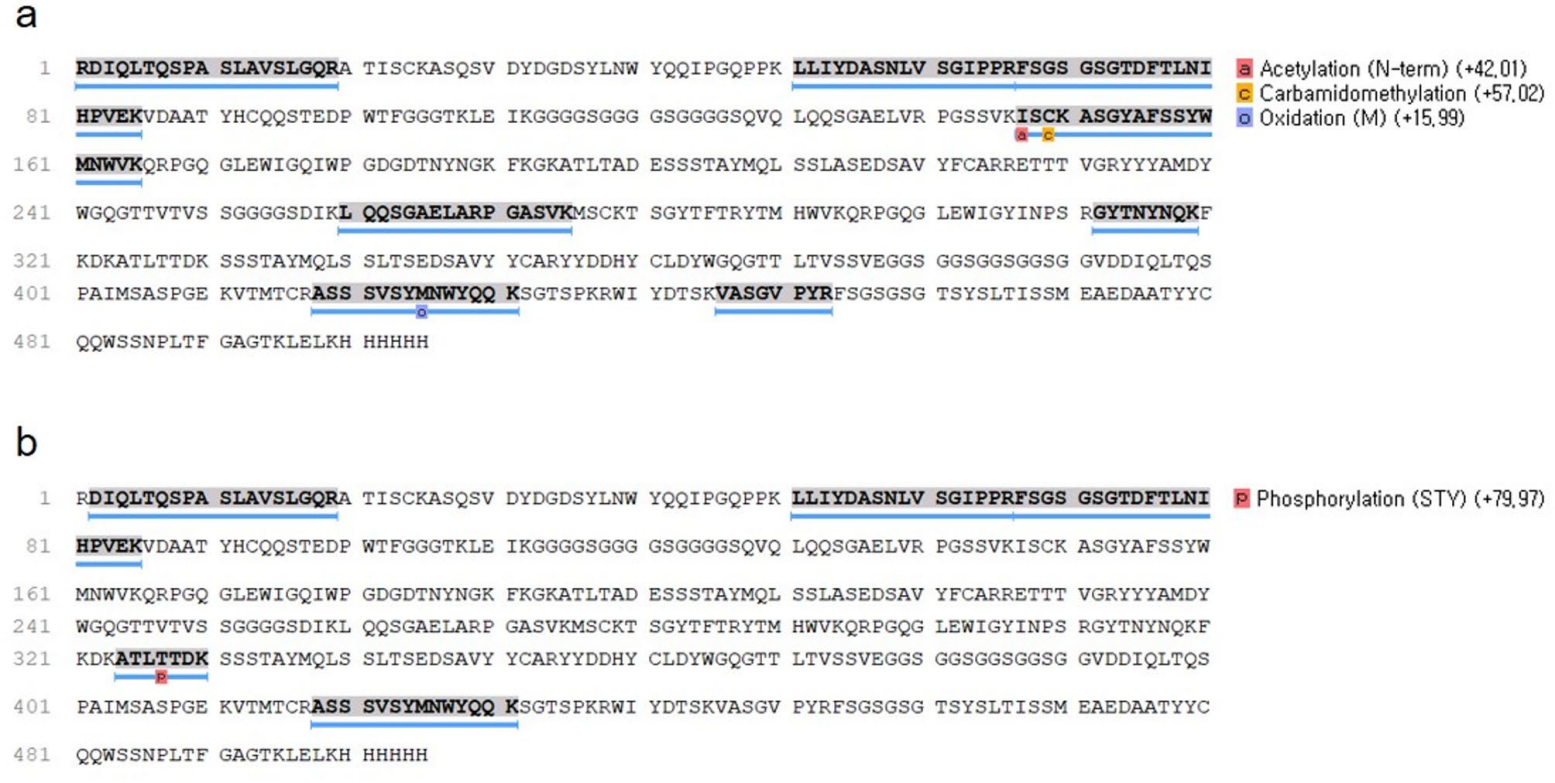
Comparison of sequence between p-blinatumomab and BLINCYTO after identification by peptide mapping. (**a**) p-blinatumomab sequence and matched coverage are shown with blue color underlining and percentage of covered sequence with detected peaks. (**b**) BLINCYTO sequence and matched coverage are shown with blue color underlining and percentage of covered sequence with detected peaks.

To compare the sequence identity of p-blinatumomab and BLINCYTO produced in CHO cells, sequence matching of p-blinatumomab was evaluated by LC-ms/ms analysis and PEAKS Software (Fig. 5). Through peptide mapping, we confirmed the matched amino acid sequences (blue underlined) between p-blinatumomab and BLINCYTO. The covered sequences with detected peaks included acetylation (N-term) (42.01%), carbamidomethylation (57.02%), oxidation (15.99%), and phosphorylation (STY) (79.97%). Therefore, this peptide mapping analysis confirms that the primary amino acid sequence of p-blinatumomab produced in *P. pastoris* (except arginine) is identical to that of BLINCYTO.

### P-blinatumomab and BLINCYTO characterization

**Fig. 6:**
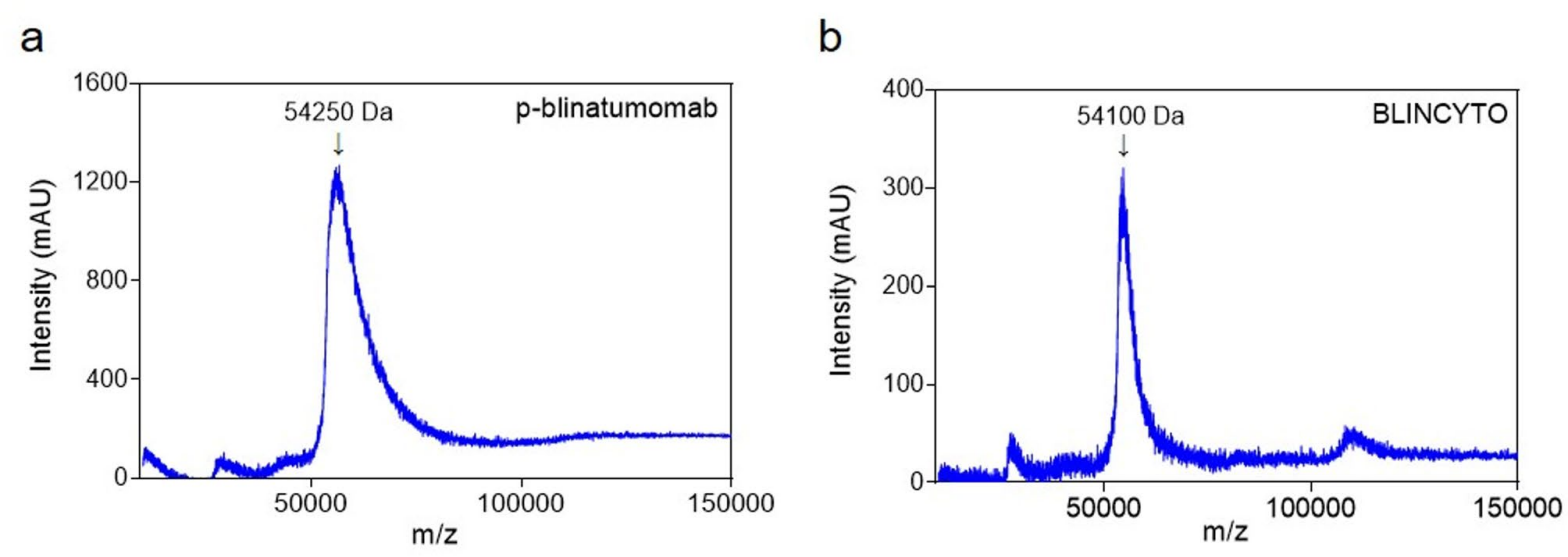
Comparison of molecular weight between p-blinatumomab and BLINCYTO. (**a**,**b**) p-blinatumomab and BLINCYTO protein molecular weights analyzed by MALDI-TOF MS. (**a**) Protein p-blinatumomab with a molecular weight of 54,250 Da. (**b**) Protein BLINCYTO with a molecular weight of 54,100 Da.

The molecular mass of the p-blinatumomab was measured using MALDI-TOF MS to confirm its molecular weight and verify its identity (Fig. 6a and b). The graph shows that the molecular weights of p-blinatumomab and BLINCYTO were 54,250 and 54,100 Da, respectively, indicating that the only difference in molecular weight corresponds to the arginine (Molecular weight of arginine: 174.2 Da).

### CD19- and CD3ε-targeting ability of p-blinatumomab

**Fig. 7:**
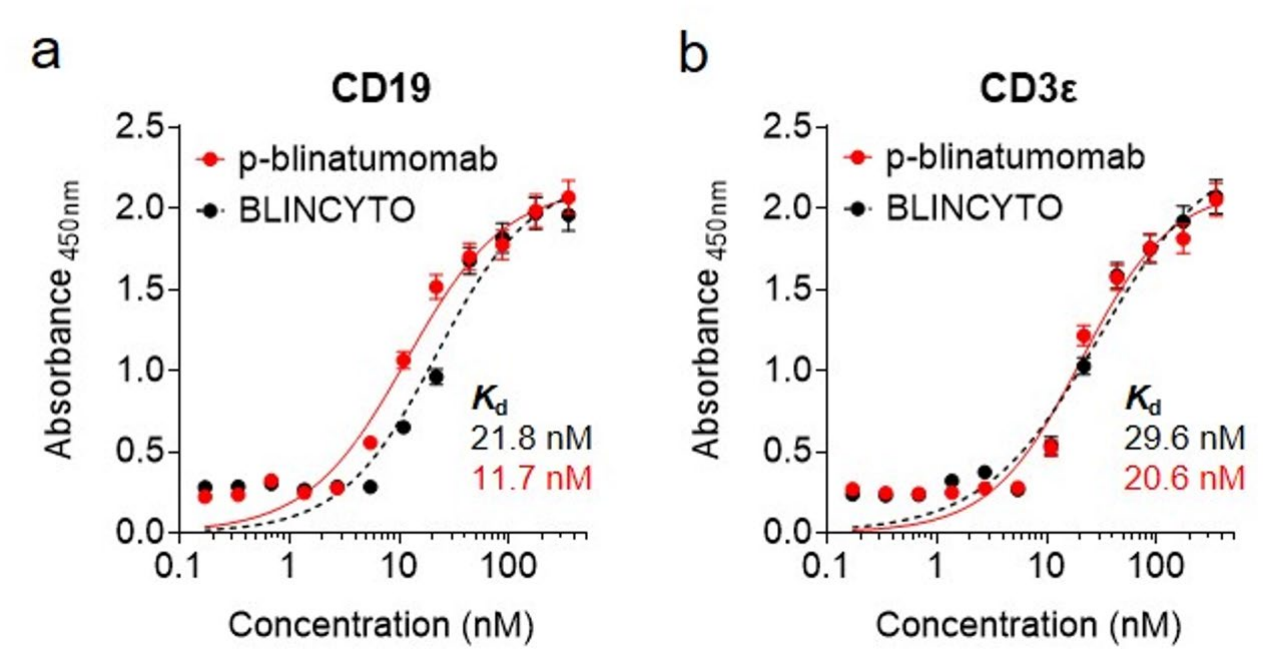
Binding affinity analysis of p-blinatumomab for CD19 and CD3ε. (**a**,**b**) Binding affinities of BLINCYTO and p-blinatumomab to (a) CD19 and (b) CD3ε using enzyme-linked immunosorbent assay (ELISA). The dissociation constants (*K*d) were calculated using non-linear regression analysis. All values are expressed as the mean ± standard deviation (SD) based on three independent experiments (*n* = 3).

To confirm the cancer-targeting ability, the CD19- and CD3ε-binding affinities of p-blinatumomab and BLINCYTO were evaluate using ELISA (Fig. 7). P-blinatumomab and BLINCYTO were diluted to 0.3–350 nM, and then incubated with the cell lines for 7 h. p-blinatumomab exhibited a stronger CD19-binding affinity (*K*_d_: 11.7 nM) than BLINCYTO (*K*_d_: 21.8 nM; Fig. 7a). However, p-blinatumomab and BLINCYTO showed similar CD3ε-binding affinities (Fig. 7b). Supplementary Figs. 1 and 2 present the dot plots and histograms for p-blinatumomab and BLINCYTO. Collectively, we confirmed that p-blinatumomab is similar and potentially superior to BLINCYTO (*K*_d_, p-blinatumomab: 20.6 nM, BLINCYTO: 29.6 nM).

**Fig. 8:**
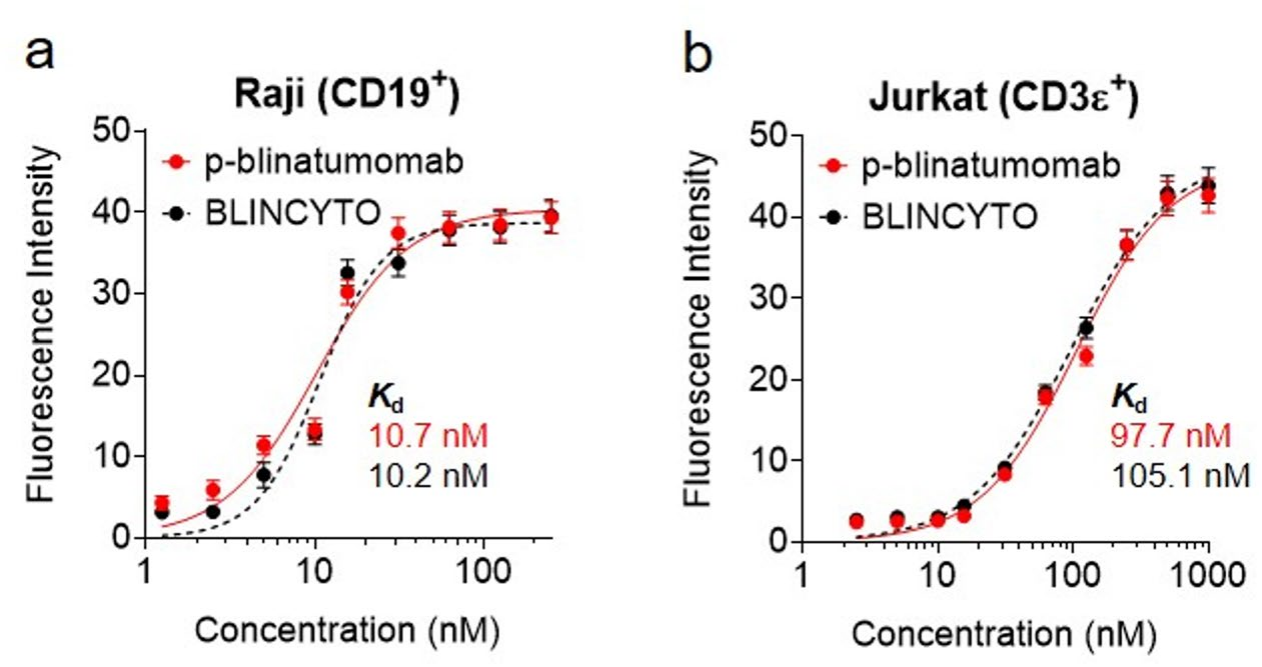
Flow cytometry analysis for evaluating CD19- and CD3ε-targeting ability of p-blinatumomab. (**a**,**b**) Binding affinities of BLINCYTO and p-blinatumomab to (**a**) CD19-overexpressing Raji cells and (**b**) CD3ε-overexpressing Jurkat cells. The dissociation constants (*K*d) were calculated using non-linear regression analysis. All values are expressed as the mean ± standard deviation (SD) based on three independent experiments (*n* = 3).

Afterwards, the CD19-binding affinities of 1.0–250 nM BLINCYTO and p-blinatumomab were compared in CD19-overexpressing Raji cell line, and their CD3ε-binding affinities were compared using 2 nM–1 µM of each protein in CD3ε-overexpressing Jurkat cell line using FCM (Fig. 8). The results indicate that the *K*_d_ of p-blinatumomab and BLINCYTO were 10.7 and 10.2 nM, respectively, in the Raji cell line (Fig. 8a). In addition, in Jurkat cell line, the *K*_d_ of p-blinatumomab and BLINCYTO were 97.7 and 105.1 nM, respectively, implying that their CD19- and CD3ε-binding abilities are similar (Fig. 8b). The dot plots and histograms are provided in Supplementary Fig. 3 and 4 for p-blinatumomab and BLINCYTO.

## Discussion

The survival rate of patients with relapsed/refractory B-cell ALL drastically declines in both children and adult groups^5^. Moreover, the mortality rate of patients with ALL living in low-SDI regions is higher than that of patients with ALL living in other regions^6^. Nonetheless, the FDA-approved blinatumomab used for treating ALL has undesirable characteristics, such as low productivity and high manufacturing costs. Antibody-based medications, such as blinatumomab, are generally manufactured in CHO cells with slow cell growth, leading to decreased productivity. A large amount of medium is required to produce adequate amounts of antibody drugs, resulting in high manufacturing costs^15^. Therefore, productivity should be enhanced to reduce costs. To overcome these disadvantages, we developed an antibody-based drug production platform using the yeast *P. pastoris*. In-house developed blinatumomab consists of anti-CD19 and anti-CD3ε scFv sequences from commercial blinatumomab drug sequence with an R amino acid added to the N-terminus. Each anti-CD19 and anti-CD3ε scFv sequence was linked by a GGGGS linker. This 6×His-tagged anti-CD19/CD3ε scFv sequence was inserted into the pHIL-S1 plasmid vector to generate recombinant blinatumomab. After analyzing the anti-CD19/CD3ε BiTE antibodies produced from two *P. pastoris* strains GS115 and KM71, GS115, which exhibits high productivity, integrity, and quality, was selected for this study. These results are consistent with the following established findings. Regarding the characteristics of GS115 and KM71, both *P. pastoris* strains GS115 and KM71 (a mutant strain derived from GS115) are commonly used for the production of secretory proteins^19^. GS115 has two genes, namely *AOX1* and *AOX2* that encode the AOX enzyme, which plays an important role in the metabolism of MeOH and the induction of protein expression^19^. *AOX1* transcription is strongly induced in the presence of MeOH in GS115 strain^19^. However, the deletion of *AOX1* is observed in strain KM71^19^. As a result, protein expression and yield remain low in the presence of MeOH, despite the use of *AOX1* as a promoter^19^. Finally, the sequence for CD19/CD3ε BiTE antibody integrated into *P. pastoris* strain GS115 was named as GS115/p-blinatumomab and #105 clone was selected. The molecular weight of p-blinatumomab protein from GS115/p-blinatumomab #105 clone was similar to that of BLINCYTO. Mass analysis and peptide mapping confirmed that the recombinant p-blinatumomab protein had the BLINCYTO sequence, with an arginine residue added to the N-terminus. The production yield of p-blinatumomab (1.2 mg/L) was 2.4 times higher than that of blinatumomab in the CHO cell expression system (Purification yield: 0.5 mg/L) based on a previous study^25^. The CD3ε-binding affinity of p-blinatumomab was 1.43 times higher than that of BLINCYTO; however, p-blinatumomab showed 1.86-fold higher CD19-binding affinity than BLINCYTO in ELISA. In FCM, p-blinatumomab specifically targeted CD19 in Raji cell line and CD3ε in Jurkat cell line, similar to BLINCYTO. Thus, p-blinatumomab effectively targets cancer antigens expressed in ALL, such as CD9 and CD3ε (Table 1).

**Table 1:** Characterization of p-blinatumomab and BLINCYTO.

Protein	Expression Yield	Binding affinity (Kd) in ELISA		Binding affinity (Kd) in FCM		Molecular weight
p-blinatumomab	1.2 mg/L	CD19	11.7 nM;	CD19	10.7 nM	54,250 Da
		CD3ɛ	20.6 nM	CD3ɛ	97.7 nM	
BLINCYTO	N/A	CD19	21.8 nM	CD19	10.2 nM	54,100 Da
		CD3ɛ	29.6 nM	CD3ɛ	105.1 nM	

*BLINCYTO* Data not disclosed by the manufacturer. *N/A* Not applicable.

To generate recombinant antibody fragments and full-length antibodies, *P. pastoris* expression system is an alternative to mammalian systems, such as CHO cells^19^. The *P. pastoris* platform can produce therapeutic recombinant proteins with *in vitro*/*vivo* efficacy as well as correct folding, stability, and solubility properties, similar to the CHO cell system^19,21^. It has been known that *P. pastoris*-produced recombinant proteins undergo PTMs, such as glycosylation and disulfide isomerization, which allow them to fold and function properly^19,20,26,27^. However, the patterns of PTMs in yeast, including N-linked glycosylation, vary substantially from those in humans, this may thus result in immunogenicity and reduced half-life^18^. In particular, *P. pastoris* is known to produce high-mannose type N-glycans, mainly occurring in the Fc regions of the antibody, and non-conventional yeast *P. pastoris* provides a customized expression platform for the production of recombinant proteins by controlling PTMs, including glycosylation^27–29^. Blinatumomab is programmed to be non-glycosylated, allowing easy entry into tumors^13^. Because our p-blinatumomab is an scFv, lacking the Fc domain, glycosylation was not observed in either p-blinatumomab or BLINCYTO by peptide mapping. High antigen binding affinity, confirmed in the ELISA and FCM, indirectly suggested that p-blinatumomab was not influenced by glycosylation in our study. In addition, the accuracy of protein folding and disulfide bond formation are also crucial issues in the production of recombinant proteins^30^. Although protein folding or folding fidelity was not measured in this study, p-blinatumomab displayed almost the same sequence and molecular weight as BLINCYTO through LC-MS.MS and MALDI-TOF analysis.

Importantly, the *P. pastoris* platform overcomes the disadvantages of CHO cell system regarding productivity and expenditure^19,20^. In a biomanufacturing reactor, *P. pastoris* is cultivated in media containing MeOH at high cell densities, leading to low-cost production^26^. MeOH as a carbon source drives the *AOX1* promoter, which is frequently used in the *P. pastoris* platform, leading to high expression of recombinant proteins and rapid growth rate^19,31,32^. In this study, we used an *AOX1*-driven MeOH induction system. According to a previous study, this *AOX1*-driven MeOH induction system enables scalability up to a demonstration scale of 1000 L fermentation^33^. MeOH is a major concern because of its flammability and toxicity during manufacturing and large-scale fermentations^32^, whereas *P. pastoris* has been recognized as safe for use following the Generally Recognized As Safe (GRAS) guidelines, regarding its biosafety^34^. To address this issue, a previous study has introduced that an *AOX1*-driven MeOH-independent induction system based on glucose–glycerol shift induction has been suggested as an alternative to the conventional MeOH-induced promoter system, which is safety due to the absence of MeOH during the protein production process^32^. Another study demonstrated that the methanol-inducible promoters, such as *AOX1* and formate dehydrogenase (*FMDH*), are activated by constantly expressing transcription factor Prm1p without MeOH, which is driven under the control of constitutive promoters, including *TEF*, *PGK*, or *GAP*^35^. Upon complete recombinant protein expression, the *P. pastoris* system secretes recombinant proteins into the culture media, resulting in a high yield, enhanced stability, and simplified purification^20,36^. Additionally, the doubling time of *P. pastoris* is 60–120 min, whereas that of CHO cells is 24 h, which ultimately improves productivity and reduces maintenance costs^19^. According to previous studies on the production yields and costs of CHO cells and *P. pastoris*, the production yield of secreting recombinant protein ACT017 was 1.8 g/L with 108-h culture time in *P. pastoris*, whereas in CHO cells, the yield was 1.0 g/L with 10-day culture time^17^. Furthermore, according to the results of the cost evaluation, ACT017 was produced at a cost of 96.44 EUR (€)/g and 222,124 €/batch using *P. pastoris*, while the production using CHO cells cost 119.13 €/g and 397,158 €/batch^17^. Consequently, *P. pastoris* showed a lower cost than CHO cells, being 1.235-fold cheaper per gram and 1.788-fold cheaper per batch^17^. With these advantages, the first FDA-approved *P. pastoris*-derived ecallantide (Kalbitor) is a representative example, which is currently used for treating hereditary angioedema, exhibiting high selectivity and rapid action onset^37^. In addition, insulin, Hepatitis B antigen, and HSA have been reported to be expressed as therapeutic proteins using the *P. pastoris*-expression system^27^.

This study is associated with some limitations. First, there was no in-house production of p-blinatumomab by the CHO cell expression system. All comparisons with CHO cells, including the cost and production yield, were based on previous studies. Second, we found that both p-blinatumomab and BLNCYTO were non-glycosylated; however, the impacts of additional differences in PTMs, including acetylation, carbamidomethylation, oxidation, and phosphorylation, on the structure and function of p-blinatumomab were not elucidated. Third, physicochemical characterization, such as disulfide bond formation and thermostability, are important information for drug bioavailability. Although we only confirmed the functionality of p-blinatumomab, further biophysical studies may be required to characterize its physicochemical properties. In addition, although we confirmed the antigen binding affinity of p-blinatumomab for CD19 and CD3ɛ, the EC_50_ or *K*a for p-blinatumomab could not be determined. Such experiments are worthwhile for therapeutic drug development. Forth, *in vivo* experiments on p-blinatumomab were not performed; therefore, *in vivo* efficacy, stability, and immunogenicity remained unmeasured.

**Fig. 9:**
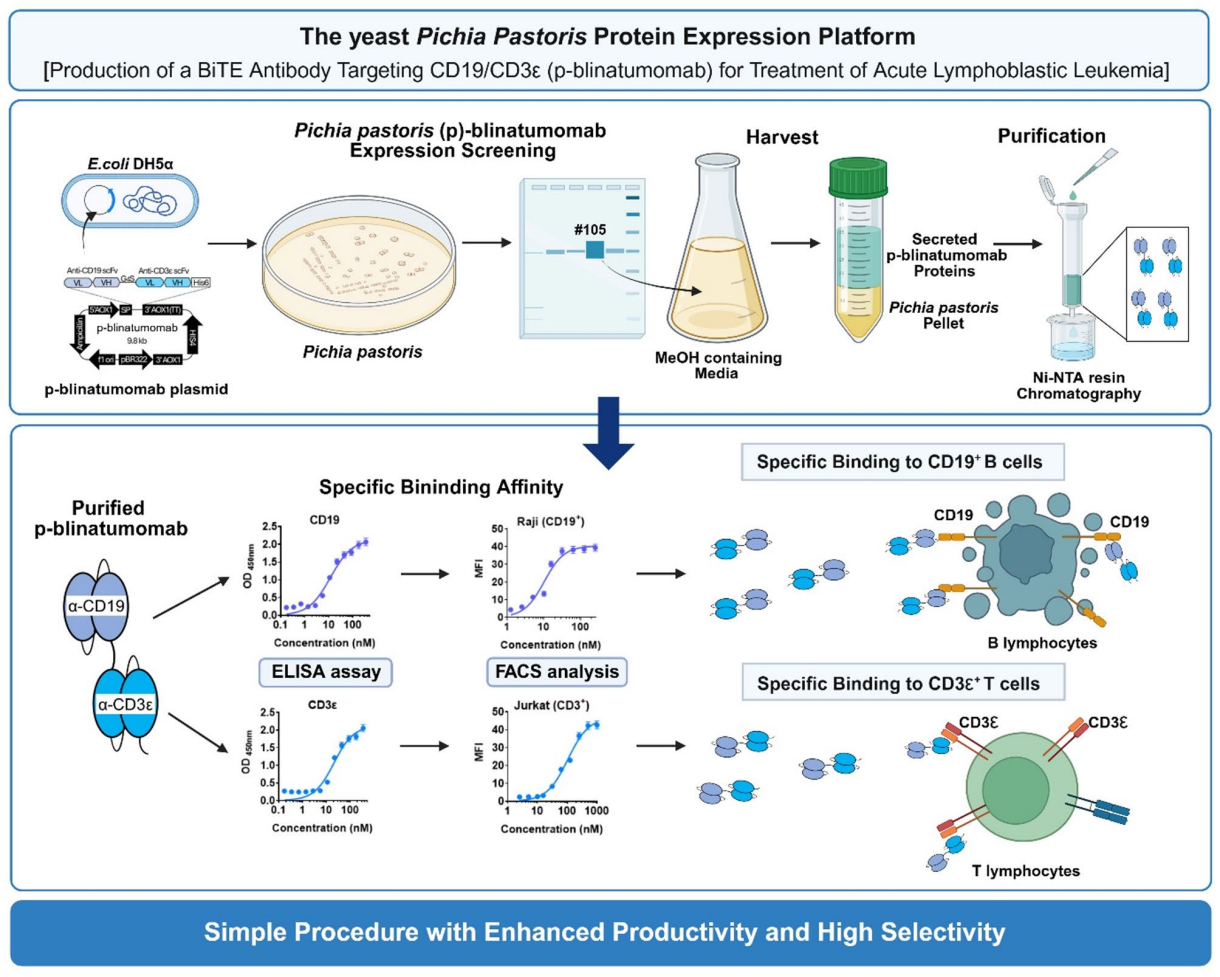
*Pichia pastoris* is a useful expression host. *P. pastoris* produces the BiTE antibody platform with high productivity, selectivity, and low costs. *P. pastoris*-produced BiTE antibody (p-blinatumomab) targeting CD19 and CD3ε could be effectively used in research, industry, or clinical settings to treat acute lymphoblastic leukemia.

In conclusion, we suggest that *P. pastoris* is a useful expression host for producing BiTE antibody platforms with high productivity, high selectivity, and low cost (Table 2). Based on this platform, our p-blinatumomab shows the potential to be used in research and clinical settings to treat ALL. We also propose that this *P. pastoris* platform can contribute to advancements in economic aspects of biopharmaceuticals (Fig. 9).

**Table 2:** Different expression systems.

	*P*. pastoris	E. coli	Mammalian cells
Secretion	**++**	**-**	**+/++**
Protein Productivity	**++**	**+**	**-**
Cost	**--**	**--**	**++**
Cost of growth medium	**-**	**-**	++ (CHO cells)
Doubling time	60–120 min	30 min	24 h (CHO cells)

Table format and data partially adapted from Gonçalves et al. (2013) and Karbalaei, M. et al. (2020) with modification.

++ high, +/++ medium to high, + medium, - low; -- very low/absent.

## Supplementary Information

Below is the link to the electronic supplementary material.

Supplementary Material 1

## Data Availability

All data used in the current study are available from the corresponding author on reasonable request. Data is provided within the manuscript or supplementary information files.
