# Supplementary material for: Production system establishment and in vitro study of CD19/CD3ε bite antibody secreted from Pichia pastoris
Source: Sci Rep. 2025 Oct 30;15:38061. doi: 10.1038/s41598-025-21889-4 (PMC12575637; doi:10.1038/s41598-025-21889-4)
Supplement: Supplementary file 1 — Supplementary Material 1 [file 41598_2025_21889_MOESM1_ESM.pdf]

Supplementary Data

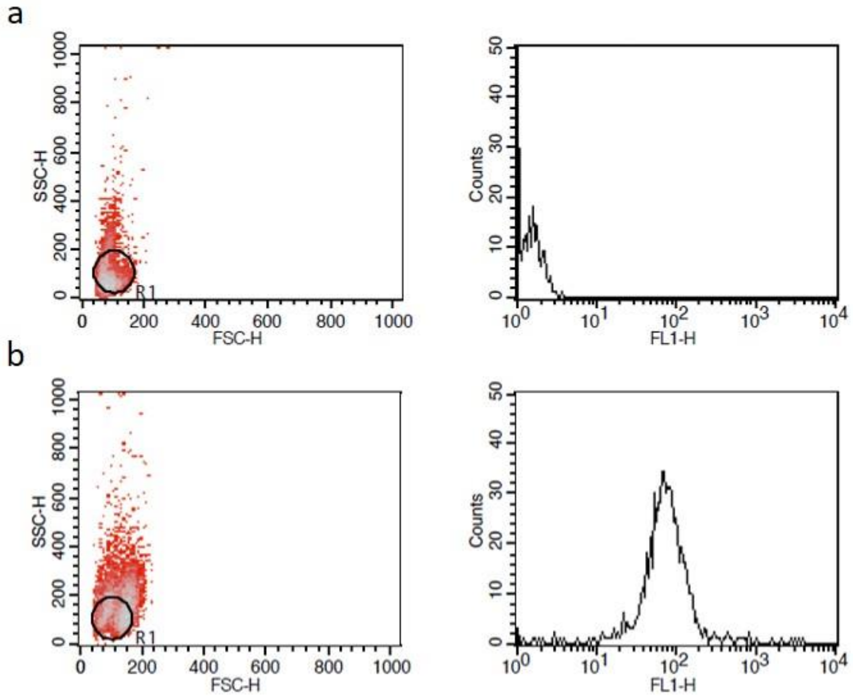

Supplementary Figure 1. Flow cytometry analysis of p-blinatumomab binding to CD19 on Raji cells.

(a) Raji cells ($CD19^+$, $CD3\varepsilon^-$) incubated without p-blinatumomab (0 nM) were analyzed by flow cytometry. The left panel shows FSC-H & SSC-H plots used to gate the lymphocyte population (R1). The right panel displays the FL1-H histogram of gated cells, indicating baseline fluorescence in the absence of p-blinatumomab. (b) Raji cells ($CD19^+$, $CD3\varepsilon^-$) treated with 250 nM p-blinatumomab were similarly analyzed. The left panel shows FSC-H & SSC-H plots and gating strategy. The right panel, in contrast for (a), depicts a pronounced rightward shift in the FL1-H histogram, demonstrating specific binding of p-blinatumomab to CD19 on Raji cells.

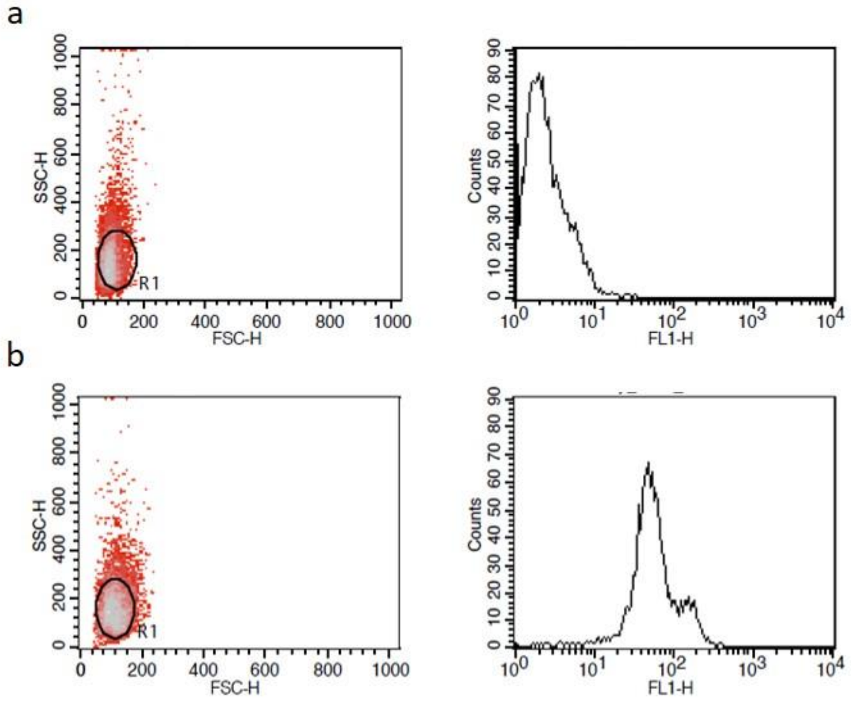

Supplementary Figure 2. Flow cytometry analysis of BLINCYTO binding to CD19 on Raji cells.

(a) Raji cells ($CD19^{+}$, $CD3\varepsilon^{-}$) incubated without BLINCYTO (0 nM) were analyzed by flow cytometry. The left panel shows FSC-H & SSC-H plots used to gate the lymphocyte population (R1). The right panel displays the FL1-H histogram of gated cells, indicating baseline fluorescence in the absence of BLINCYTO. (b) Raji cells ($CD19^{+}$, $CD3\varepsilon^{-}$) treated with 250 nM BLINCYTO were similarly analyzed. The left panel shows FSC-H & SSC-H plots and gating strategy. The right panel, in contrast for (a), depicts a pronounced rightward shift in the FL1-H histogram, demonstrating specific binding of BLINCYTO to CD19 on Raji cells.

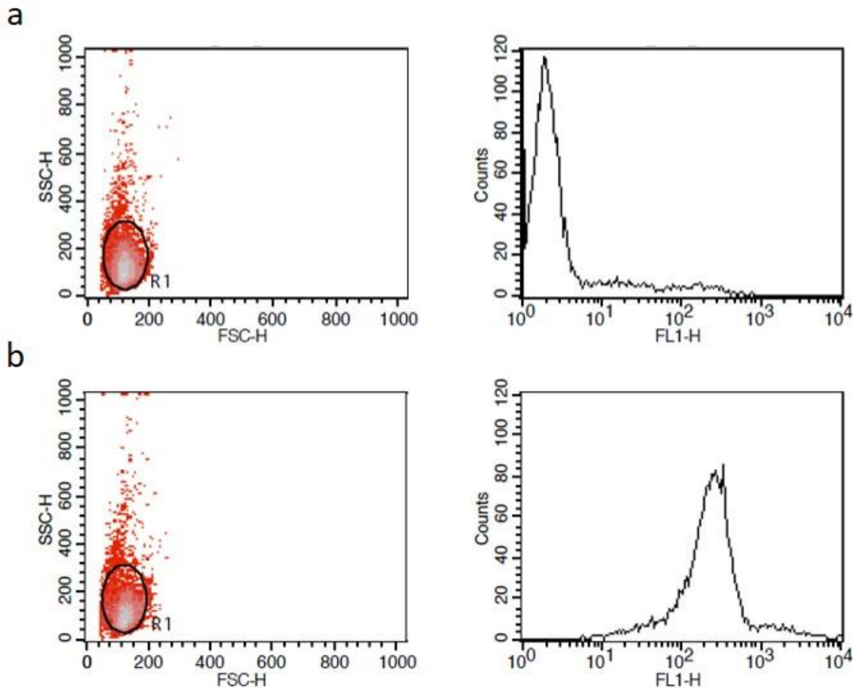

Supplementary Figure 3. Flow cytometry analysis of p-blinatumomab binding to CD3ε on Jurkat cells.

(a) Jurkat cells ($CD19^-$, $CD3ε^+$) incubated without p-blinatumomab (0 nM) were analyzed by flow cytometry. The left panel shows FSC-H & SSC-H plots used to gate the lymphocyte population (R1). The right panel displays the FL1-H histogram of gated cells, indicating baseline fluorescence in the absence of p-blinatumomab. (b) Jurkat cells ($CD19^-$, $CD3ε^+$) treated with 1μM p-blinatumomab were similarly analyzed. The left panel shows FSC-H & SSC-H plots and gating strategy. The right panel, in contrast for (a), depicts a pronounced rightward shift in the FL1-H histogram, demonstrating specific binding of p-blinatumomab to CD3ε on Jurkat cells.

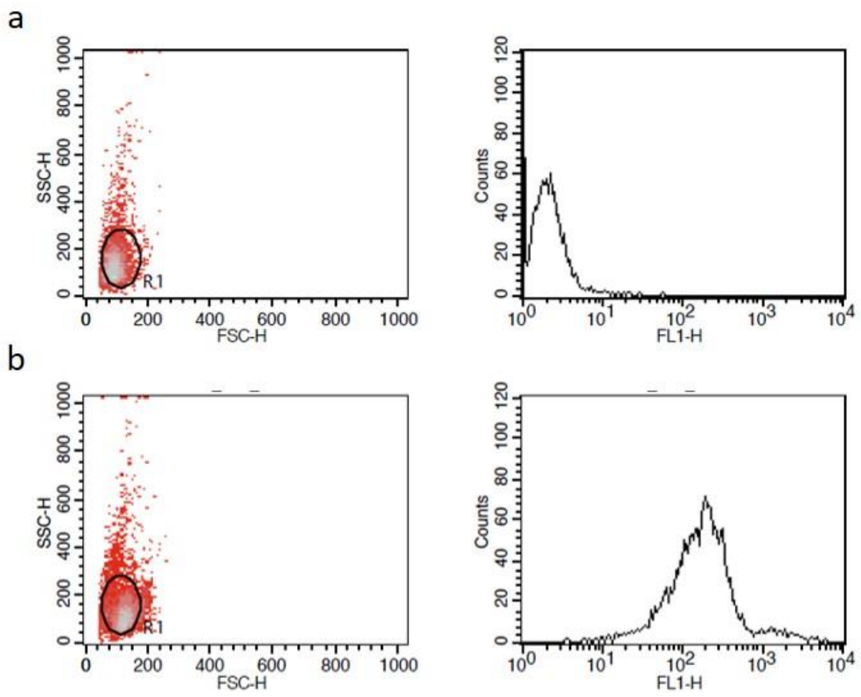

Supplementary Figure 4. Flow cytometry analysis of BLINCYTO binding to CD3ε on Jurkat cells.

(a) Jurkat cells ($CD19^-$, $CD3\varepsilon^+$) incubated without BLINCYTO (0 nM) were analyzed by flow cytometry. The left panel shows FSC-H & SSC-H plots used to gate the lymphocyte population (R1). The right panel displays the FL1-H histogram of gated cells, indicating baseline fluorescence in the absence of BLINCYTO. (b) Jurkat cells ($CD19^-$, $CD3\varepsilon^+$) treated with 1μM BLINCYTO were similarly analyzed. The left panel shows FSC-H & SSC-H plots and gating strategy. The right panel, in contrast for (a), depicts a pronounced rightward shift in the FL1-H histogram, demonstrating specific binding of BLINCYTO to CD3ε on Jurkat cells.

Supplementary Information 1.

[Inserted amino acid sequence]

RDIQLTQSPASLAVSLGQRATISCKASQSVDYDGDSYLNWYQQIPGQPPKLLIYDASNLVSGIPPRFSGSGSGTDFTLNIHPVEKVDA
ATYHCQQSTEDPWTFGGGTKLEIKGGGGSGGGGSGGGGSQVQLQQSGAELVRPGSSVKISCKASGYAFSSYWMNWVKQRPGQG
LEWIGQIWPGDGDTNYNGKFKGKATLTADESSSTAYMQLSSLASEDSAVYFCARRETTTVGRYYYAMDYWGQGTTVTVSSGGG
GSDIKLQQSGAELARPGASVKMSCKTSGYTFTRYTMHWVKQRPGQGLEWIGYINPSRGYTNYNQKFKDKATLTTDKSSSTAYMQ
LSSLTSEDSAVYYCARYYDDHYCLDYWGQGTTLTVSSVEGGSGGSGGSGGSGGVDDIQLTQSPAIMSASPGEKVTMTCRASSSVS
YMNWYQQKSGTSPKRWIYDTSKVASGVPYRFSGSGSGTSYSLTISSMEAEDAATYYCQQWSSNPLTFGAGTKLELKHHHHHH

[Anti-CD19 scFv]

RDIQLTQSPASLAVSLGQRATISCKASQSVDYDGDSYLNWYQQIPGQPPKLLIYDASNLVSGIP
PRFSGSGSGTDFTLNIHPVEKVDAATYHCQQSTEDPWTFGGGTKLEIKGGGGSGGGGSGGGG
SQVQLQQSGAELVRPGSSVKISCKASGYAFSSYWMNWVKQRPGQGLEWIGQIWPGDGDTN
YNGKFKGKATLTADESSSTAYMQLSSLASEDSAVYFCARRETTTVGRYYYAMDYWGQGTT
VTVSS

[Linker]

GGGGS

[Anti-CD3ε scFv]

DIKLQQSGAELARPGASVKMSCKTSGYTFTRYTMHWVKQRPGQGLEWIGYINPSRGYTNYN
QKFKDKATLTTDKSSSTAYMQLSSLTSEDSAVYYCARYYDDHYCLDYWGQGTTLTVSSVEG
GSGGSGGSGGSGGVDDIQLTQSPAIMSASPGEKVTMTCRASSSVSYMNWYQQKSGTSPKRW
IYDTSKVASGVPYRFSGSGSGTSYSLTISSMEAEDAATYYCQQWSSNPLTFGAGTKLELK

[6x His tag]

HHHHHH

Supplementary Information 2.

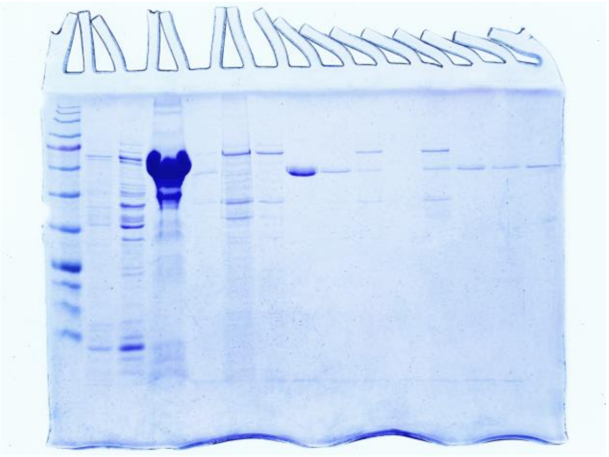

A full-length SDS-PAGE gel image in Figure 2a.

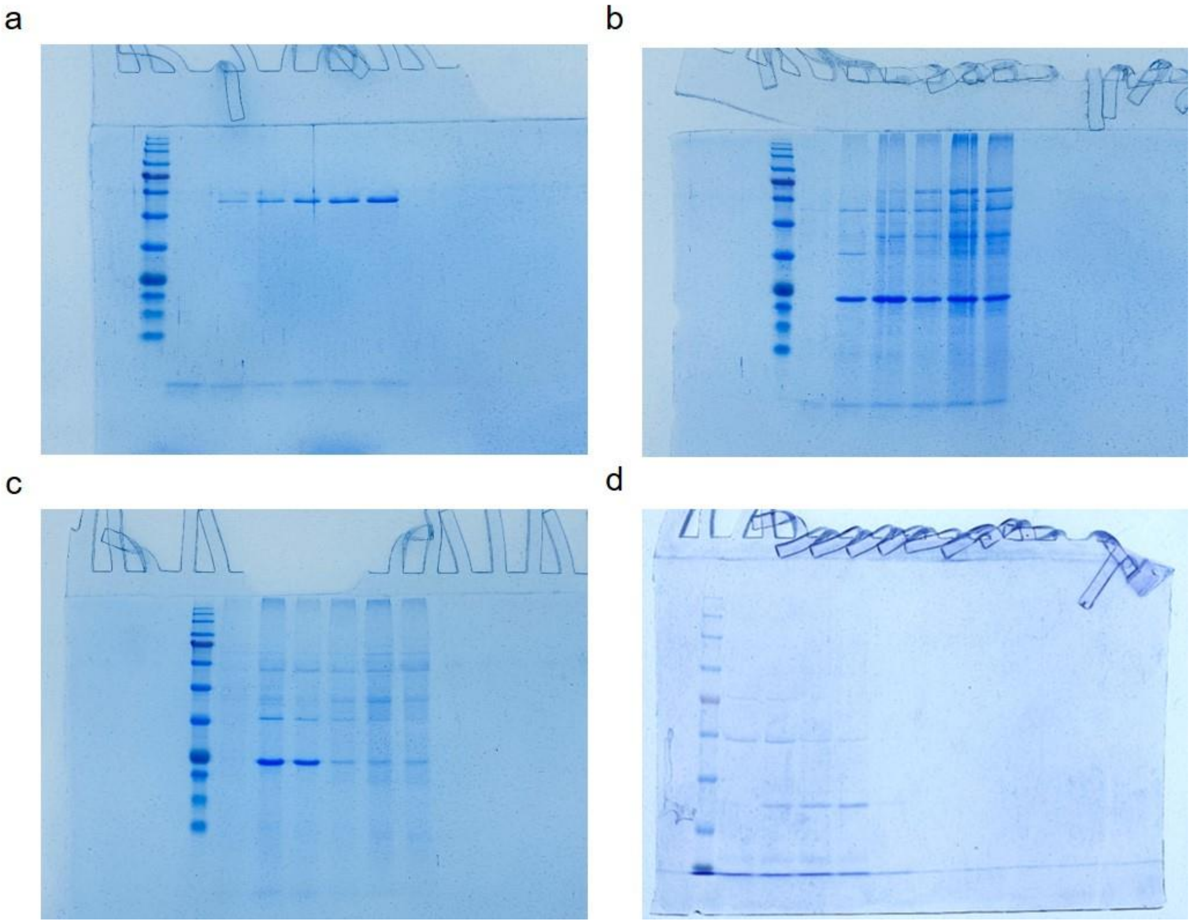

Full-length SDS-PAGE gel images in Figure 3.

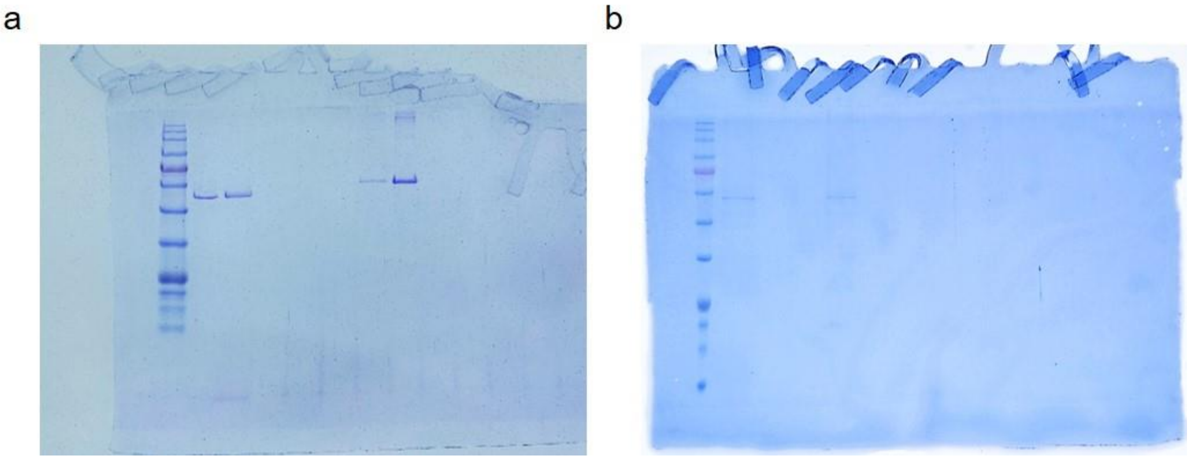

Full-length SDS-PAGE gel images in Figure 4.
